# Mental health reported in adult invasive home mechanical ventilation through a tracheostomy: A scoping review

**DOI:** 10.1016/j.ijnsa.2022.100110

**Published:** 2022-11-24

**Authors:** Martin Locht Pedersen, Charlotte Handberg, Pia Dreyer

**Affiliations:** a Department of Public Health, Aarhus University, Bartholins Allé 2, 8000 Aarhus C, Denmark; b Forensic Mental Health Research Unit Middelfart (RFM), Department of Regional Health Research, Faculty of Health Science, University of Southern Denmark, Østre Hougvej 70, 5500 Middelfart, Denmark; c Psychiatric Department Middelfart, Mental Health Services in the Region of Southern Denmark, Østre Hougvej 70, 5500 Middelfart, Denmark; d National Rehabilitation Centre for Neuromuscular Diseases, Kongsvang Allé 23, 8000 Aarhus C, Denmark; e Department of Anaesthesiology and Intensive Care, Aarhus University Hospital, Palle Juul-Jensens Boulevard 99, 8200 Aarhus N, Denmark; f Department of Global Public Health and Primary Care, University of Bergen, Årstadveien 17, 5020 Bergen, Norway

**Keywords:** Chronic respiratory failure, Home care services, Invasive mechanical ventilation, Mental health nursing, Respiratory insufficiency, Review study, Tracheostomy treatment, Well-being

## Abstract

**Background:**

Although people receiving invasive home mechanical ventilation through a tracheostomy are facing both physical and mental health challenges, healthcare services often focus mainly on physical symptoms. To ensure well-functioning treatment and care for people receiving tracheostomy ventilation in a home setting, their mental health needs to be promoted and seen as an integral part of their health in general.

**Objective:**

This scoping review aimed to provide a summary of the current knowledge on the mental health of people receiving invasive home mechanical ventilation through a tracheostomy.

**Design:**

A scoping review of published and gray literature based on the framework developed by Arksey and O'Malley and refined by the JBI was performed. The Preferred Reporting Items for Systematic Reviews and Meta-analyses Extension for Scoping Reviews checklist was used for reporting the findings.

**Methods:**

A literature search was conducted by two researchers independently in the PubMed, CINAHL and PsycINFO databases. Additional searches for gray literature were conducted in Google, Google Scholar, websites of selected organisations, and the reference lists of included studies. The software system Covidence was used in the study selection process. For critical appraisal, the Mixed Methods Appraisal Tool was used.

**Results:**

Thirteen studies were included in this review, of which six used qualitative, six quantitative, and one mixed methods. The majority of studies were authored in Europe (*n* = 10), followed by the Americas (*n* = 2) and the Western Pacific (*n* = 1). Mental health was investigated both directly and indirectly (61.5% vs. 38.5%). Categorizing the reported mental health outcomes, we found that emotional well-being was reported widely across the studies (*n* = 13), while psychological well-being (*n* = 5) and social well-being (*n* = 4) were less widely reported.

**Conclusions:**

The mental health of people receiving home tracheostomy ventilation has received some scholarly attention. A heterogeneity of mental health outcomes was reported in the literature with emotional well-being being an important mental health area both in relation to the sub-components positive affect and quality of life appraisal. Mental health outcomes in relation to psychological well-being and social well-being were fragmented and only sparsely investigated.

## What is already known about the topic?

Home mechanical ventilation is a versatile and complex treatment, and the number of people receiving this treatment is increasing worldwide.

Even though people receiving invasive home mechanical ventilation through a tracheostomy encounter physical and mental health challenges, treatment and care focus on physical symptoms.

## What this paper adds

The research area generally appears very heterogenous, and reporting on mental health outcomes is based on both direct and indirect investigations.

We found that home mechanical ventilation therapy is associated with both positive and negative outcomes, mostly in relation to emotional well-being, followed by psychological well-being and social well-being.

## 1 Introduction

Mental health problems are common, and mental illness is projected to become a leading cause of burden to the world by 2030 (Mathers and Loncar, 2006). However, good mental health is not merely absence of illness. The World Health Organization (2004) describes good mental health as ‘a state of well-being in which the individual realizes his or her own abilities, can cope with the normal stresses of life, can work productively and fruitfully, and is able to make a contribution to his or her community’ (p. 59). The World Health Organization (2004) report highlights that mental health needs to be promoted and should be seen as an integral part of people's health in general. Home mechanical ventilation can be experienced very differently by people receiving this treatment (Dreyer et al., 2010a; Dyrstad et al., 2013). Major psychological, physiologic, social, and existential challenges characterize the changed life situation facing people receiving home mechanical ventilation (Lindahl and Kirk, 2019; Ørtenblad et al., 2019). Although people receiving invasive home mechanical ventilation through a tracheostomy are facing both physical and mental health challenges, healthcare services and extant research have focused mainly on physical symptoms. Therefore, a knowledge gap exists concerning the mental health of these people. The purpose of the present scoping review was therefore to close this knowledge gap in relation to people receiving invasive home mechanical ventilation through a tracheostomy.

## 2 Background

Home mechanical ventilation is a versatile and complex treatment area provided outside a hospital setting for people with chronic respiratory failure (Hind et al., 2017; Lewarski and Gay, 2007; Simonds, 2016). Both invasive (tracheostomy) and non-invasive (mask) ventilator treatment can be used in the home setting (MacIntyre et al., 2016; Park and Suh, 2020). In recent years, the number of people receiving home mechanical ventilation has increased in some countries (Nasiłowski et al., 2015; Povitz et al., 2018). The prevalence of such treatment is estimated at 6.6 (range: 0.1–17.0) per 100,000 population in Europe (Lloyd-Owen et al., 2005), of whom 13% receive tracheostomy ventilation. The prevalence of people receiving home mechanical ventilation varies across countries and is estimated at 12.9 per 100,000 population in Canada (Rose et al., 2015), with 18% receiving tracheostomy ventilation; in Australia and New Zealand, the estimated prevalence is 9.9–12.0 per 100,000 population, with only 3.1% receiving tracheostomy ventilation (Garner et al., 2013); and in South Korea, the estimated prevalence is 9.3 per 100,000 population, with 62.8% receiving tracheostomy ventilation (Kim et al., 2019).

Healthcare services for people receiving home mechanical ventilation are organised in very different ways across countries; in Denmark, home mechanical ventilation is organised by three university hospital units (Winther et al., 2020); in Sweden, by 50 clinics that manage the treatment of people receiving home mechanical ventilation (Laub and Midgren, 2007); in Portugal, by 30 home mechanical ventilation units in public hospitals (Mineiro et al., 2020); and in Canada, by a combination of public and private community and institutional providers that make up 152 units providing home mechanical ventilation services (Rose et al., 2015). An organised healthcare structure for users of home mechanical ventilation is critical to underpinning meaningfulness and good quality of life for even profoundly disabled people (Stuart and Weinrich, 2004), like those who are invasively ventilated through a tracheostomy at home. In a Swedish quality-of-life-study, people receiving home mechanical reported good perceived health, despite severe physical limitations (Markström et al., 2002).

People receiving invasive home mechanical ventilation through a tracheostomy often have severe physical disabilities requiring respiratory treatment and extensive care. They also often need personal help performed by relatives or a team of formal or informal personal assistants (Winther et al., 2020; Dybwik et al., 2011; Israelsson-Skogsberg and Lindahl, 2017; MacLaren et al., 2019; Schaepe and Ewers, 2018). Treatment in the home setting requires competence in terms of both treatment and care and a focus on the user's individual needs. Indeed, several researchers have documented the importance of personal assistants’ competence as a prerequisite for successful collaboration (Dreyer et al., 2010a; Briscoe and Woodgate, 2010; Dyrstad et al., 2013; Laakso et al., 2011). The personal assistant should also be able to cater to the users’ mental needs to enhance their understanding and help them cope with daily challenges, thereby promoting their well-being as described by the World Health Organization (2004).

A review of seven qualitative studies showed that people receiving home mechanical ventilation report better quality of life than people who receive no such assistance (Ørtenblad et al., 2019). The same positive effect on health-related quality of life was reported in a systematic review of 26 quantitative studies (MacIntyre et al., 2016). However, the changed life situation with invasive home mechanical ventilation through a tracheostomy may also impair mental health. The negative mental health effects may include fear (Briscoe and Woodgate, 2010), worries (Dreyer et al., 2010b; Briscoe and Woodgate, 2010; Dyrstad et al., 2013), and insecurity (Dale et al., 2018; Dyrstad et al., 2013) and may make the people fight for autonomy (Briscoe and Woodgate, 2010). In a systematic review of nine qualitative and eight quantitative studies focusing on tracheostomy-ventilated people and their personal assistants in varying settings, a range of mostly negative experiences concerning well-being and quality of life were reported (Nakarada-Kordic et al., 2018).

In this scoping review, we summarize current knowledge on the mental health of adults receiving invasive home mechanical ventilation through a tracheostomy. To the best of our knowledge, no scoping review has previously examined this issue. The present study may therefore provide highly relevant data to improve healthcare services and care solutions and define future research needs in the field.

## 3 Methods

### 3.1 Aims

The aims of this scoping review were (a) to characterize available studies on the mental health of adults receiving invasive home mechanical ventilation through a tracheostomy and (b) to identify the reported mental health outcomes used in this setting. The following research questions guided this review: What is the focus in the studies, and how has mental health been investigated? What characterises mental health outcomes?

### 3.2 Design

This scoping review was based on the methodological framework developed by Arksey and O'Malley (2005) and refined by the JBI (Peters et al., 2020). In line with this framework, the following steps were undertaken: (1) identifying the research questions, (2) identifying relevant studies, (3) selecting study, (4) charting the data, and (5) collating, summarizing and reporting the results. The Preferred Reporting Items for Systematic Reviews and Meta-analyses Extension for Scoping Reviews checklist was used for reporting the findings (Page et al., 2021; Tricco et al., 2018).

### 3.3 Population, concept and context

The following elements were used to identify the research question: *population, concept,* and *context* (Peters et al., 2020): (a) *Population*: Adults (≥18 years old) with chronic respiratory failure needing invasive home mechanical ventilation through a tracheostomy; (b) *Concept*: Mental health was defined as any reported mental health outcome in accordance with the World Health Organization definition cited above. Mental health outcomes were categorised according to Keyes's (2014) three components of mental health: (1) Emotional well-being, (2) psychological well-being and (3) social well-being (Table 1); (c) *Context*: Home mechanical ventilation was defined in accordance with international studies (Garner et al., 2013; Lloyd-Owen et al., 2005) as invasive mechanical ventilation through a tracheostomy outside a hospital setting, delivered at home or in a long-term care environment, with daily variation in time of ventilation through a tracheostomy depending on the individual's needs.

**Table 1:** Components and sub-components of mental health according to Keyes (2014).

**Components**	**Sub-components**
Emotional well-being	Positive Affect and Judgements of Quality of Life
Psychological well-being	Self-Acceptance, Personal Growth, Purpose in Life, Environmental Mastery, Autonomy and Positive Relations with Others
Social well-being	Social Acceptance, Social Growth, Social Contribution, Social Coherence and Social Integration

### 3.4 Search strategy

The literature search was conducted using an established methodology aimed to find both published and gray literature. According to the JBI (Peters et al., 2020), a three-step process was performed by two researchers, and the process was verified by a librarian. Firstly, an initial search was conducted in PubMed and CINAHL databases. This was followed by a process of identifying keywords, subject headings, and synonyms to capture any potential resources from the databases. Secondly, a search with an inclusive approach was conducted in PubMed, CINAHL, and PsycINFO. In this step, a well-refined block search strategy was employed using all identified keywords, subject headings, and synonyms combined into the search string with Boolean operators OR/AND/NOT (De Brún and Pearce-Smith, 2014). The search strategy was prepared according to the conditions of the individual database (see Table 2 for a detailed example in CINAHL). The time span was from 01 January 2010 to 31 December 2020 (the final search date; updated on 24 August 2022) to ensure a contemporary knowledge base in a field of significant development; no language limitations were used in the search. Thirdly, a search for additional relevant studies was made using the reference lists of the included articles and reviews and guidelines identified through the search process. For additional gray literature, Google, Google Scholar, and websites of selected organisations (e.g., Muscular Dystrophy Association [mda.org] and The Danish Rehabilitation centre for Neuromuscular Diseases [rcfm.dk]) were hand searched by the researchers (Aromataris and Riitano, 2014).

**Table 2:** Search strategy with keywords, subject headings and synonyms combined with Boolean operators (OR/AND/NOT) in the database CINAHL.

**Block 1:**	Home mechanical ventilation OR Home mechanical ventilator OR Ventilation at home OR Ventilator support at home OR Home tracheostomy ventilation OR Invasive mechanical ventilation at home OR Domiciliary ventilation OR Long-term mechanical ventilation OR HMV OR IHMV OR LTMV OR Prolonged mechanical ventilation at home OR Home prolonged mechanical ventilation
**Block 2:**	AND MH "Psychological Processes and Principles+" OR MH "Behavior and Behavior Mechanisms+" OR MH "Behavioral and Mental Disorders+" OR MH "Disciplines, Tests, Therapy, Services+" OR MH “Mental Health” OR MH “Wellness” OR MW “Psychosocial Factors” OR Mental health OR Quality of life OR Psychiatr* OR Psycholog* OR Psychosocial* OR Social* OR Emotion* OR Well-being* OR Wellbeing*
**Block 3:**	NOT TI Child* OR TI Pediatric* OR TI Paediatric* OR TI Neonat* OR TI Preterm* OR TI Newborn* OR TI Premature* OR TI Infant* OR TI Intensive care unit* OR TI ICU

Limitations: Peer-reviewed; Published date: 01 January 2010–24 August 2022.

### 3.5 Study selection criteria and process

Studies were included according to the following criteria: (a) Studies reporting on the mental health (*concept*) of adults (*population*) receiving invasive home mechanical ventilation through a tracheostomy (*context*); (b) based on all types of qualitative, quantitative, and mixed method studies. The exclusion criteria were the following: (a) studies without reported mental health outcomes in a setting of home mechanical ventilation; (b) studies that did not distinguish between intubated and tracheostomised people or used mixed participant categories in other ways (e.g., invasive and non-invasive); or (c) studies without empirical data or where full-text articles could not be obtained.

The study selection process took place in two stages according to the above inclusion and exclusion criteria, and the software system Covidence was used. First, two researchers (MLP and PD) screened the studies’ titles and abstracts independently. All the relevant studies were included and proceeded onto the second stage. Any studies on which the researchers did not agree whether they should be included or excluded proceeded onto the second stage of the screening process before a final decision was made. Second, two researchers (MLP and PD) made a full-text screening of the studies independently to identify studies to include in this scoping review. Disagreements regarding eligibility of studies were discussed between the researchers until consensus or the support of the third researcher (CH) was obtained, if required.

### 3.6 Quality appraisal

According to the methodological framework used in this scoping review, assessment of the methodological quality is not compulsory (Arksey and O'Malley, 2005; Peters et al., 2020). However, researchers have stated that a lack of quality assessment in scoping reviews makes the results more challenging to interpret and use (Brien et al., 2010; Grant and Booth, 2009). Therefore, the included studies (*N* = 13) were critically appraised by two researchers (MLP and PD (*n* = 7); MLP and CH (*n* = 6)) using the Mixed Methods Appraisal Tool (Hong et al., 2018; Tricco et al., 2018). The final appraisal was based on a subsequent discussion of the results. The Mixed Methods Appraisal Tool consists of two screening questions for all types of study designs, followed by five questions targeting the particular category of study design (Hong et al., 2018).

### 3.7 Data charting and analysis

A data charting form inspired by the JBI (Peters et al., 2020) was used in the data extraction process. To be able to answer the questions in this review, the following data were extracted from each article: (a) author(s), year of publication, country of origin, and journal; (b) study aim and design; (c) home setting; (d) participants; (e) key findings of mental health and how mental health was investigated. Data extraction was initiated and conducted by the first researcher (MLP) and supervised by the other researchers (PD and CH). Following the data extraction process, the data were summarised to answer the research questions (Arksey and O'Malley, 2005; Peters et al., 2020).

## 4 Results

### 4.1 Studies included

**Fig. 1:**
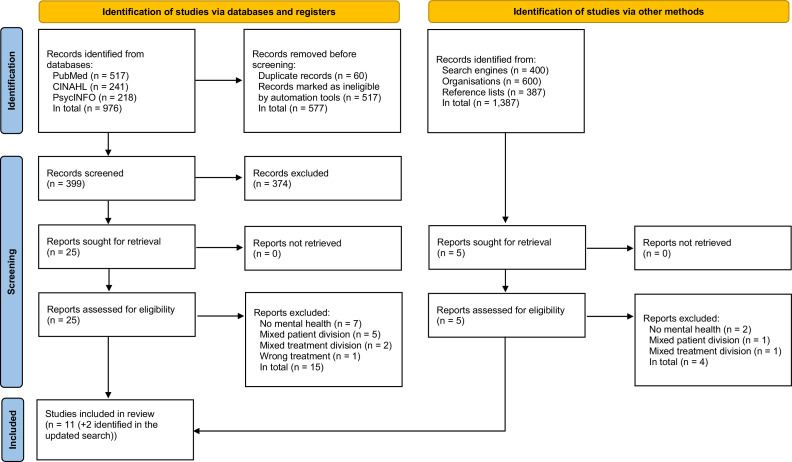
PRISMA flow diagram of the study selection process.

Initially, a total of 2363 studies were identified. After duplication removal and screening for relevance, 30 studies were assessed for eligibility. Another 19 studies were excluded following application of the inclusion criteria, and 11 studies were included. The updated search identified two additional studies. Finally, 13 studies were included in this review. The study selection process and information about exclusion are illustrated in Fig. 1 (Page et al., 2021).

#### 4.1.1 Methodological quality appraisal

All included studies were assessed to give a positive response to the two screening questions in the Mixed Methods Appraisal Tool. The methodological quality was assessed to be high in nine studies (Dreyer et al., 2010b; Briscoe and Woodgate, 2010; Huttmann et al., 2015; Jacobs et al., 2021; Jacobs et al., 2020; Klingshirn et al., 2022; Nelissen et al., 2019; Valko et al., 2020; Yamaguchi and Suzuki, 2013) and medium in four studies (Andersen et al., 2017; Dale et al., 2018; Dyrstad et al., 2013; Rousseau et al., 2011) (Table 3).

**Table 3:** Included studies reporting the mental health of tracheostomy-ventilated people.

**Author(s), year, country and journal**	**Aim**	**Design**	**Home setting**	**Participants**	**Investigation of the mental health**	**Key findings**	**Summary of critical appraisal**
Andersen et al. (2017); Denmark; Spinal Cord Series and Cases.	To describe the Danish population of tetraplegics with a phrenic nerve pacer implant and compare them to a group of respiratorily impaired, mechanical ventilator-dependent tetraplegics in order to elucidate possible differences imposed by the pacer.	Cross-sectional design, comparable case series using medical records and structured follow-up interview.	Lived in their own home (private) (*n* = 7).	Seven participants (7 male); >18 years old; diagnosis: tetraplegia; all receiving tracheostomy ventilation; all had a personal helper 24 hours per day; experience of ventilation at home from 2.5 to 24.6 years. (7 participants who had a phrenic nerve pacer implant).	Mental health investigated directly due to quality of life focus.	No significant differences were found when comparing quality of life with individuals who had implantation of a phrenic nerve pacer; the participants seemed to have a better quality of life than a standard population (SF36 mental health summary).	Medium quality. Uncertain if the sample strategy is relevant for addressing the research question, or if the risk of non-response bias is low.
Briscoe and Woodgate (2010); Canada; Qualitative Health Research.	To explore the subjective meaning of the experience of transition from spontaneous breathing to reliance on long-term mechanical ventilation.	Hermeneutic phenomenological design using semi-structured, in-dept, individual interviews; follow-up interview; and questionnaire.	Lived in an apartment or house (*n* = 6) or in a long-term care residence (*n* = 5).	Eleven participants (2 male); 40–88 years old; various diagnoses: neurological/degenerative or neuromuscular disease (*n* = 7), thoracic wall deformity (*n* = 3), quadriplegia (*n* = 1); all were invasively ventilated via a tracheostomy, and 7 had utilized non-invasive treatment in the years prior to tracheostomy; most participants were ventilated 24 h daily; experience of ventilation at home from 2 to 21 years.	Mental health investigated indirectly.	The essence of the users’ transition experience was sustaining self despite psychological, physical and spiritual challenges, including a struggle to overcome circumstances that posed a threat to the self; choosing not to die meant living in a changed life situation, and the ventilator technology holding one onto life (a sense of life going on); users of long-term mechanical ventilation struggled for their right to self-determination and autonomy in relation to the health professionals and the health system; a paradox is experienced by the users starting long-term mechanical ventilation due to renewed energy or vigor concurrently with new restrictions; they want to recreate a home in which they could be themselves, but it was difficult for those who should live in a long-term care facility; the experience of successfully dealing with ventilator problems that gave the users a sense of control over their machine; it is a life with uncertainty, and with fear and worry about the future.	High quality.
Dale et al. (2018); Canada; Annals of the American Thoracic Society.	To elucidate barriers to, and facilitators of, transition to home mechanical ventilation from the perspective of Canadian ventilator-assisted individuals and their family caregivers.	Descriptive, qualitative design using semi-structured, individual interviews; ventilator-assisted individual–family caregiver dyad interviews; and deductive contents analysis.	Lived at home (*n* = 10).	Ten participants (7 male); 21–77 years old; various diagnoses: neuromuscular disease (*n* = 4), spinal cord injury (*n* = 2), amyotrophic lateral sclerosis (*n* = 2), restrictive lung disease (*n* = 1), muscular dystrophy (*n* = 1); continuous (24 h) or part-time ventilation; all were tracheostomy ventilated and could have had non-invasive ventilation prior to tracheostomy; full- or parttime ventilation; experience of ventilation at home from 3 to 24 months. (9 participants receiving non-invasive ventilation and 14 participants were family caregivers).	Mental health investigated indirectly.	Home mechanical ventilation advanced their autonomy, independence (they had to have the capacity to address malfunctioning equipment) and quality of life; a range of people from the family and community were an indispensable resource and their support reduced the insecurity; it was important to develop positive relations with personal support workers.	Medium quality. Uncertain if the interpretation of results is sufficiently substantiated by data.
Dreyer et al., (2010b); Denmark; International Journal of Qualitative Studies on Health and Well-being.	To study life experiences of people living with Duchenne's muscular dystrophy, home mechanical ventilation, and physical impairment.	Phenomenological hermeneutic approach using individual, narrative interviews.	Lived at home (on their own, with their parents or in shared living with others) (*n* = 18) or in an institution (*n* = 1).	Nineteen participants (19 male); 21–40 years old; diagnosis: Duchenne muscular dystrophy; all tracheostomized ventilated and ten had utilized non-invasive treatment prior to tracheostomy; all had personal assistance 24 h a day; experience from 3 to 18 years.	Mental health investigated indirectly.	Home mechanical ventilation gave the users the capacity to live an active life; they considered themselves to be the same person they had always been, even though there were some activities they could not do; they dreamed about a normal family life and wanted to live as normal as possible; they did not feel psychologically impaired but could feel excluded from society because of poor accessibility; they described themselves as independent human beings; a positive outlook on life was important for the users of home mechanical ventilation; lack of social and physical accessibility leads to loneliness; the youth were described as difficult, worrying and lonely; you just have to be patient and suddenly you will succeed.	High quality.
Dyrstad et al. (2013); Norway; Journal of Clinical Nursing.	To describe the self‐reported life situation of users totally dependent on home mechanical ventilation after tracheotomy and to identify factors associated with user satisfaction.	Descriptive, explorative qualitative design using individual, semi-structured interviews and qualitative contents analysis.	Lived at home (with family or in their own flat) (*n* = N*/*A) or in a nursing home (*n* = N*/*A).	Six participants (3 male); 37–78 years old; a range of diagnoses: N/D; all dependent on home mechanical ventilation through a tracheostomy around the clock; experience with ventilation at home from 2 to over 10 years.	Mental health investigated directly due to a user satisfaction focus.	They had a need to participate in organization of treatment and care and activities of daily life, and in making their own decisions; and being taken seriously was important; the users living at home expressed satisfaction with their life situation and social life seemed to be better; confidence in caregiver continuity and level of competences, nursing home and community were important; it is important to feel safe when one's health and strength are impaired; some worried about safety if they did not trust the caregiver.	Medium quality. Uncertain if the qualitative data collection methods are adequate to address the research question, or if there is coherence between qualitative data sources, collection, analysis and interpretation.
Huttmann et al. (2015); Germany; Respiration.	To establish detailed information on living conditions and health-related quality of life in patients with invasive home mechanical ventilation.	Clinical investigation using a cross-sectional design with questionnaires in addition to structured and semi,- structured individual interviews.	Lived at home (private) (*n* = 13), in a nursing home specialized for home mechanical ventilation (*n* = 12) or in a community with an ambulatory nursing service (*n* = 7).	Thirty-two participants (15 male); 31–67 years old; various diagnoses: chronic obstructive pulmonary disease (*n* = 11), amyotrophic lateral sclerosis (*n* = 6), chronic lung disease (*n* = 4), spinal cord injury (*n* = 3), hereditary muscular metabolic diseases (*n* = 2), Curschmann-Steinert myotonic dystrophy (*n* = 2), overlap syndrome with chronic obstructive pulmonary disease and coexisting obesity hypoventilation syndrome (*n* = 2), Duchenne muscular dystrophy (*n* = 1), destroyed lung (*n* = 1); all invasively ventilated through a tracheostomy and 9 had non-invasive treatment prior to tracheostomy; between 2 and 24 h of ventilation daily; experience of ventilation at home from 2 to 132 months.	Mental health investigated directly due to a quality of life focus.	Neuromuscular patients tended to have a better quality of life than patients with lung diseases (SRI summary scale scores); living in a private home compared to living in nursing facilities did not influence the quality of life (SRI scores); the quality of life was highly heterogeneous; living situation did not influence the quality of life.	High quality. Uncertain if the risk of non-response bias is low.
Jacobs et al. (2021); Israel; Journal of the American Medical Directors Association.	To describe the profile of patients receiving invasive PMV at home compared with those in HLTC.	Descriptive observational study using structured interviews and medical records.	N/D	Forty-six participants (26 male); mean age: 53.8 ± 21.3 years old; various diagnosis: degenerative neurological causes (*n* = 27), chronic lung disease (*n* = 12), acute neurological causes (*n* = 8), other (*n* = 4), post sepsis (*n* = 3); all invasively ventilated through a tracheostomy; most participants were ventilated 24 h daily; median (IQR) duration of ventilation at home: 51.2 months (28.0, 199).	Mental health investigated directly due to a depression focus.	People receiving prolonged mechanical ventilation were less likely to be depressive when comparing with those in hospital long term care.	High quality.
Jacobs et al. (2020); Israel; Journal of the American Medical Directors Association.	To describe the mood, well-being, distressing symptoms and attitudes toward prolonged ventilation among prolonged mechanical ventilation patients.	Observational cross-sectional study using medical records, questionnaire and structured interviews.	Lived at home (*n* = 40) or in long-term acute care (*n* = 22).	Sixty-two participants (34 male); mean age: 61.7 ± 20.8 years old; various diagnoses: neurologic degenerative (*n* = 27), chronic lung disease (*n* = 18), neurologic acute (*n* = 10), post sepsis (*n* = 9), other (*n* = 8), heart disease (*n* = 3); all invasively ventilated through a tracheostomy; most participants were ventilated >18 h daily; mean duration of ventilation experience at home: 36.6 months (interquartile range (IQR) 10.8–114.1).	Mental health investigated directly due to a mood and well-being focus.	Few people receiving prolonged mechanical ventilation reported distressing symptoms, depression and impaired well-being, and mostly less at home than in long-term acute care.	High quality. Uncertain if the risk of nonresponse bias is low.
Klingshirn et al. (2022); Germany; BMC Nursing	To examine quality of care as a multifaceted construct (i.e., clinical and perceived quality of care) that considered the different living conditions in outpatient intensive care with a mixed-methods approach.	Mixed-methods convergent parallel design using semi-structure and structured interviews and online survey.	Lived at home (private) (*n* = 20) or in shared living community (*n* = 17).	Thirty-seven participants (24 male); mean age: 55.02±16.96 years old; various diagnoses: neuromuscular disorder (*n* = 16), central nervous system disease (*n* = 8), chronic obstructive pulmonary disease (*n* = 5), spinal cord paralysis (*n* = 4), pneumonia (*n* = 2), Post-operative complications (*n* = 2); all invasively ventilated through a tracheostomy; most participants received continuous ventilation; mean duration of ventilation experience at home: 5.22 years (range: 0.14–32.96). (9 participants receiving non-invasive ventilation).	Mental health investigated directly due to a quality of life focus.	No significant differences were found when comparing quality of life with individuals who had non-invasive ventilation (SRI Summary Score).	High quality.
Nelissen et al. (2019); Germany; Central European Journal of Nursing and Midwifery.	To examine the experiences and life circumstances of people with home mechanical ventilation via a tracheostomy.	A qualitative approach using a ground theory methodology; problem-centered interviews.	Lived at home (alone, with life companions, spouse or parents) (*n* = 7), in intensive care residential community (*n* = 7), in specialised inpatient care facility (*n* = 4) or in an assisted living facility (alone) (*n* = 2).	Twenty participants (13 male); 23–90 years old; various diagnoses: chronic obstructive pulmonary disease (*n* = 8), motor neuron disease (*n* = 4), cardiac decompensation (*n* = 1), congenital myopathy of unclear genesis (*n* = 1), mucopolysaccharidosis type VI (*n* = 1), morbid obesity (*n* = 1), progressive muscular dystrophy (*n* = 1), phrenic nerve palsy with diaphragmatic elevation (*n* = 1), incomplete cross-section paralysis (*n* = 1), complete cross-section paralysis (*n* = 1); all invasively ventilated through a tracheostomy; most participants were ventilated 24 h daily; experience of ventilation at home from 2 month to 24 years.	Mental health investigated indirectly.	At the start, they did not feel being a human being and instead felt stigmatized; they got more strength and energy during the day and got greater quality of life; they were able to get on with their lives, which they must embrace; they prepared to accept major restrictions; not every experience boosted the participants’ desire to continue living; trust in the nursing staff was important; the opposite led to a fear of loss and the positive attitude towards the staff might decrease; contact with other people through a computer, which made it possible to take active part in life again; living autonomously again, to regain independence and making decisions about daily routines was important; self-determination and independence, increased the opportunity to accept the current life circumstances; happy about every day they felt well, but this did not mean that they were content with everything.	High quality.
Rousseau et al. (2011); France; Journal of Neurology.	To compare quality of life of amyotrophic lateral sclerosis and locked-in-syndrome patients with invasive mechanical ventilation to amyotrophic lateral sclerosis and locked-in-syndrome patients without invasive mechanical ventilation.	Cross-sectional design using questionnaire.	N/D	Twelve participants; mean age: 49 years old (range: 24–64); various diagnoses: amyotrophic lateral sclerosis (*n* = 8), locked-in-syndrome (*n* = 4); all invasively ventilated through a tracheostomy; mean duration of ventilation experience at home: 43.5 months (range: 2–204). (22 participants receiving non-invasive ventilation).	Mental health investigated directly due to a quality of life focus.	Invasive mechanical ventilation for patients who accept tracheotomy did not affect quality of life; no significant differences were found when comparing quality of life between individuals with and without mechanical invasive ventilation.	Medium quality. Uncertain if the sample is representative of the target population, or if the statistical analysis is appropriate to answer the research question.
Valko et al. (2020); Hungary; BMC Pulmonary Medicine	To evaluate quality of life change patterns 6 months after initiation of home mechanical ventilation in patients suffering from chronic respiratory failure using patient-reported outcomes.	Prospective observational cohort study using questionnaire.	Lived at home (*n* = 14).	Fourteen participants; various diagnoses: N/D; all invasively ventilated through a tracheostomy; hours of ventilation daily: 17.9 ± 6.5; experience of ventilation at home from 0 to 6 months. (52 participants receiving non-invasive ventilation).	Mental health investigated directly due to a quality of life focus.	A profound effect of home mechanical ventilation on quality of life; the quality of life was dependent on initial diagnoses and baseline quality of life; interface was not associated with improvement in quality of life.	High quality.
Yamaguchi and Suzuki (2013); Japan; International Journal of Qualitative Studies on Health and Well-being.	To better understand the process by which patients with Duchenne muscular dystrophy who are on home mechanical ventilation and who prefer an independent life in their communities arrived at the goal of independent living (i.e., choosing to live at home in Japan instead of in special sanatoriums that provide sufficient support and care).	Grounded theory approach using semi-structured interviews.	Lived at home (*n* = 1).	One participant (1 male); between 22 and 57 years old; diagnosis: Duchenne muscular dystrophy; invasively ventilated through a tracheostomy. (20 participants receiving invasive (not distinguishable) or non-invasive ventilation).	Mental health investigated indirectly.	It was not perceived as a life to live in the sanatorium unless it was just the physical disability from which he needed to be freed; it was life-enriching to move out of the hospital.	High quality.

N/D, not defined. N/A, not available. SF36, Short Form (36) Health Survey. SRI, Severe Respiratory Insufficiency Questionnaire. Contents in parentheses refer to elements in the studies not included in this scoping review.

#### 4.1.2 Description of studies

The included studies are summarised in Table 3. Of the 13 studies included, 10 were authored in Europe (Andersen et al., 2017; Dreyer et al., 2010b; Dyrstad et al., 2013; Huttmann et al., 2015; Jacobs et al., 2021; Jacobs et al., 2020; Klingshirn et al., 2022; Nelissen et al., 2019; Rousseau et al., 2011; Valko et al., 2020). Outside Europe, two studies were authored in the Americas (Briscoe and Woodgate, 2010; Dale et al., 2018) and one in the Western Pacific (Yamaguchi and Suzuki, 2013). Eleven out of the 13 studies included people receiving tracheostomy ventilation in their own homes (Andersen et al., 2017; Dreyer et al., 2010b; Briscoe and Woodgate, 2010; Dale et al., 2018; Dyrstad et al., 2013; Huttmann et al., 2015; Jacobs et al., 2020; Klingshirn et al., 2022; Nelissen et al., 2019; Valko et al., 2020; Yamaguchi and Suzuki, 2013). Of these, seven studies included additional people living in home settings other than private homes (e.g., in long-term care) (Dreyer et al., 2010b; Briscoe and Woodgate, 2010; Dyrstad et al., 2013; Huttmann et al., 2015; Jacobs et al., 2020; Klingshirn et al., 2022; Nelissen et al., 2019). The two remaining studies did not define the home setting (Jacobs et al., 2021; Rousseau et al., 2011).

Seven studies were published in medical journals (Andersen et al., 2017; Dale et al., 2018; Huttmann et al., 2015; Jacobs et al., 2021; Jacobs et al., 2020; Rousseau et al., 2011; Valko et al., 2020), three in public health journals (Dreyer et al., 2010b; Briscoe and Woodgate, 2010; Yamaguchi and Suzuki, 2013), and three in nursing journals (Dyrstad et al., 2013; Klingshirn et al., 2022; Nelissen et al., 2019)). Within the investigation time span (2010–22), an average of one study per year was published, although no studies were published for three of the years (2012, 2014 and 2016). More than half of the studies (*n* = 7; 53.9%) were published from 2017 to 2022 (Andersen et al., 2017; Dale et al., 2018; Jacobs et al., 2021; Jacobs et al., 2020; Klingshirn et al., 2022; Nelissen et al., 2019; Valko et al., 2020).

Five out of the six qualitative studies were based on interview (Dreyer et al., 2010b; Dale et al., 2018; Dyrstad et al., 2013; Nelissen et al., 2019; Yamaguchi and Suzuki, 2013), and one was based on both interview and questionnaire (Briscoe and Woodgate, 2010). Of the six quantitative studies, two were based on questionnaires (Rousseau et al., 2011; Valko et al., 2020), three were based on medical records and interview (Andersen et al., 2017; Jacobs et al., 2021; Jacobs et al., 2020), and one was based on questionnaire and interview (Huttmann et al., 2015). The remaining mixed method study was based on interview and an online survey (Klingshirn et al., 2022). In total, the included studies contained findings based on 277 people receiving invasive home mechanical ventilation through a tracheostomy: 90 females and 147 males with various diagnoses. The diagnoses were mostly described as neurodegenerative (*n* = 54) (Jacobs et al., 2021; Jacobs et al., 2020) and chronic lung disease (*n* = 34) (Huttmann et al., 2015; Jacobs et al., 2021; Jacobs et al., 2020). In relation to the included participants, two studies did not report sex information (Rousseau et al., 2011; Valko et al., 2020). Likewise, two studies did not report diagnosis information (Dyrstad et al., 2013; Valko et al., 2020).

### 4.2 Mental health

Five studies investigated mental health indirectly (Dreyer et al., 2010b; Briscoe and Woodgate, 2010; Dale et al., 2018; Nelissen et al., 2019; Yamaguchi and Suzuki, 2013) (Table 3). These studies were qualitative. Their purpose was not to investigate or examine the mental health of the people receiving tracheostomy ventilation at home but rather to examine ‘the experiences and life circumstances of people with HMV [home mechanical ventilation] via a tracheostomy’ (p. 1103) (Nelissen et al., 2019) or ‘to explore the subjective meaning of the experience of transition from spontaneous breathing to reliance on LTMV [long-term mechanical ventilation]’ (p. 57) (Briscoe and Woodgate, 2010). Given these purposes, some key findings demonstrate insights into the users’ mental health. Eight studies investigated mental health directly; this included quality of life (Andersen et al., 2017; Huttmann et al., 2015; Klingshirn et al., 2022; Rousseau et al., 2011; Valko et al., 2020), user satisfaction (Dyrstad et al., 2013), and mood and well-being (Jacobs et al., 2021; Jacobs et al., 2020).

Table 4 summarises the key findings of the mental health outcomes according to Keyes's (2014) three mental health components (Table 1). As shown, various mental health outcomes were reported across the studies, and outcomes were both positive and negative. In the present review, both positive and negative outcomes were considered findings. This demonstrates the range of the reported mental health outcomes in the versatile and complex treatment area of home mechanical ventilation. All studies reported mental health outcomes in relation to emotional well-being, while five studies reported mental health outcomes in relation to psychological well-being (Dreyer et al., 2010b; Briscoe and Woodgate, 2010; Dale et al., 2018; Dyrstad et al., 2013; Nelissen et al., 2019) and four in relation to social well-being (Dreyer et al., 2010b; Dale et al., 2018; Dyrstad et al., 2013; Nelissen et al., 2019). In the following, each of the three components of mental health are reviewed.

**Table 4:** Mental health outcomes according to Keyes's (2014) three components of mental health.

**Components**	**Sub-components**	**References**	**Example of reported key findings**
Emotional well-being (*n* = 13)	Positive Affect (*n* = 7)	Dreyer et al., (2010b), Briscoe and Woodgate (2010), Dyrstad et al. (2013), Jacobs et al. (2021), Jacobs et al. (2020), Nelissen et al. (2019), Yamaguchi and Suzuki (2013)	The users living at home expressed satisfaction with their life situation and social life seemed to be better (Dyrstad et al., 2013).
	Judgements of Quality of Life (*n* = 7)	Andersen et al. (2017), Dale et al. (2018), Huttmann et al. (2015), Klingshirn et al. (2022), Nelissen et al. (2019), Rousseau et al. (2011), Valko et al. (2020)	No significant differences were found when comparing quality of life with individuals who had implantation of a phrenic nerve pacer (Andersen et al., 2017).
Psychological well-being (*n* = 5)	Self-Acceptance (*n* = 1)	Dreyer et al., (2010b)	They considered themselves to be the same person they had always been, even though there were some activities they could not do (Dreyer et al., 2010b).
	Personal Growth (*n* = 1)	Briscoe and Woodgate (2010)	The essence of the users’ transition experience was sustaining self despite psychological, physical and spiritual challenges, including a struggle to overcome circumstances that posed a threat to the self (Briscoe and Woodgate, 2010).
	Purpose in Life (*n* = 1)	Nelissen et al. (2019)	They were able to get on with their lives, which they must embrace (Nelissen et al., 2019).
	Environmental Mastery (*n* = 3)	Briscoe and Woodgate (2010), Dyrstad et al. (2013), Nelissen et al. (2019)	The experience of successfully dealing with ventilator problems that gave the users a sense of control over their machine (Briscoe and Woodgate, 2010).
	Autonomy (*n* = 3)	Briscoe and Woodgate (2010), Dale et al. (2018), Nelissen et al. (2019)	Home mechanical ventilation advanced their autonomy (Dale et al., 2018).
	Positive Relations with Others (*n* = 3)	Dale et al. (2018), Dyrstad et al. (2013), Nelissen et al. (2019)	Trust in the nursing staff was important; the opposite led to a fear of loss and the positive attitude towards the staff might decrease (Nelissen et al., 2019).
Social well-being (*n* = 4)	Social Acceptance (*n* = 2)	Dale et al. (2018), Dyrstad et al. (2013)	It was important to develop positive relations with personal support workers (Dale et al., 2018).
	Social Growth (*n* = 0)		
	Social Contribution (*n* = 1)	Nelissen et al. (2019)	Contact with other people through a computer, which made it possible to take active part in life again (Nelissen et al., 2019).
	Social Coherence (*n* = 0)		
	Social Integration (*n* = 2)	Dreyer et al., (2010b), Nelissen et al. (2019)	They did not feel psychologically impaired but could feel excluded from society because of poor accessibility (Dreyer et al., 2010b).

n, number of publications.

#### 4.2.1 Emotional well-being

Mental health in relation to emotional well-being was widely reported across the studies, even when not directly investigated. This suggests that it is an important area of mental health for people receiving invasive home mechanical ventilation through a tracheostomy.

Seven studies reported on the sub-component *positive affect* in relation to emotional well-being (Dreyer et al., 2010b; Briscoe and Woodgate, 2010; Dyrstad et al., 2013; Jacobs et al., 2021; Jacobs et al., 2020; Nelissen et al., 2019; Yamaguchi and Suzuki, 2013). The invasive tracheostomised ventilation at home gave people an interest in the lived life (Dreyer et al., 2010b; Briscoe and Woodgate, 2010; Dyrstad et al., 2013). Being ventilated kept life going, and they could live an active life (Dreyer et al., 2010b; Nelissen et al., 2019), but the renewed energy was accompanied by new restrictions that could affect the mood (Briscoe and Woodgate, 2010; Jacobs et al., 2021; Jacobs et al., 2020; Nelissen et al., 2019). A worrying life was a description that was reported several times. Living with home tracheostomy ventilation is a life with worry about the future (Briscoe and Woodgate, 2010); some worried about their safety if they did not trust the personal assistant (Dyrstad et al., 2013). Moreover, people receiving home mechanical ventilation during their youth described this time as worrying, beyond difficult, and lonely (Dreyer et al., 2010b). Those people who lived at home (private) reported having more life energy and satisfaction with the changed life situation than those who lived, for example, in a long-term care facility or in an institution (Briscoe and Woodgate, 2010; Dyrstad et al., 2013; Jacobs et al., 2020; Yamaguchi and Suzuki, 2013). This may be explained by the fact that independence was reported as important in the person's life (Dreyer et al., 2010b; Nelissen et al., 2019).

Likewise, seven studies reported on the sub-component *judgements of quality of life* (Andersen et al., 2017; Dale et al., 2018; Huttmann et al., 2015; Klingshirn et al., 2022; Nelissen et al., 2019; Rousseau et al., 2011; Valko et al., 2020). Quality of life has been heterogeneously investigated across the studies, which makes them difficult to compare. However, no studies reported decreased quality of life of people receiving home mechanical ventilation through a tracheostomy because of the treatment and care itself. Several studies reported an increased quality of life, with quality being increased both at the individual level (Dale et al., 2018; Nelissen et al., 2019; Valko et al., 2020) and compared with a general population (Andersen et al., 2017). Quality of life was reported as better among people with neuromuscular diseases than among people with lung diseases (Huttmann et al., 2015). However, increased quality of life was not reported in studies comparing tracheostomy-ventilated people with people receiving other treatments (Andersen et al., 2017; Klingshirn et al., 2022; Rousseau et al., 2011; Valko et al., 2020) or among different home settings (Huttmann et al., 2015). Quality of life among people appeared to differ much, and outcome seems to be closely associated with their diagnosis (Huttmann et al., 2015; Valko et al., 2020).

#### 4.2.2 Psychological well-being

Of the five studies that reported on mental health in relation to psychological well-being, four reported on the sub-component *environmental mastery* (Briscoe and Woodgate, 2010; Dale et al., 2018; Dyrstad et al., 2013; Nelissen et al., 2019). Here, the importance of being able to handle the responsibilities in life that come with receiving mechanical ventilation and care was reported, such as decision-making and being taken seriously (Dyrstad et al., 2013). Three studies reported on the sub-component *positive relations with others* (Dale et al., 2018; Dyrstad et al., 2013; Nelissen et al., 2019). Here, trusting relationships with family and personal assistants were reported as important to the tracheostomy-ventilated people. Likewise, three studies reported on the sub-component *autonomy* (Briscoe and Woodgate, 2010; Dale et al., 2018; Nelissen et al., 2019). Autonomy was ambiguously reported across the studies, which indicates that autonomy may be highly individual. Living autonomously was generally important to people receiving home tracheostomy ventilation. However, whereas mechanical ventilation advanced the autonomy of some people (Dale et al., 2018), others had to fight for their right to autonomy in relation to the health professionals and the healthcare system (Briscoe and Woodgate, 2010).

One study reported on the sub-components *self-acceptance, personal growth,* and *purpose in life*. This indicated that in relation to psychological well-being, these elements were not researched individually in the studies. People were generally self-accepting and considered themselves to be the same person they had always been, even though they were unable to do certain activities (Dreyer et al., 2010b). Personal growth was necessary to overcome circumstances that posed a threat to the self, such as psychological, physical, and spiritual challenges (Briscoe and Woodgate, 2010). Despite the changed life situation, people receiving home tracheostomy ventilation could see a purpose, in life as they were able to get on with their lives and embrace it (Nelissen et al., 2019).

#### 4.2.3 Social well-being

With only four studies, social well-being is the component of mental health reported least widely across the studies. These studies investigated mental health in relation to social well-being indirectly; none of the studies reported outcomes on the sub-components *social growth* and *social coherence*. Two studies reported on the sub-component *social acceptance* (Dale et al., 2018; Dyrstad et al., 2013). It was important to develop positive relations and collaborate with personal assistants. Social acceptance, however, was contingent on confidence in the continuity of the relation and the level of the carer's competencies (Dale et al., 2018; Dyrstad et al., 2013). Likewise, two studies reported on the sub-component *social integration* (Dreyer et al., 2010b; Nelissen et al., 2019). Here, the importance of getting positive support from the community was reported to be important, because feeling excluded from society because of poor accessibility led to loneliness (Dreyer et al., 2010b); tracheostomy-ventilated people could feel stigmatised, especially in the beginning of the tracheostomy trajectory (Nelissen et al., 2019). Only one study reported on *social contribution* and found that contact with other people through a computer made it possible to take an active part in life again (Nelissen et al., 2019). As with some of the sub-components of psychological well-being, the scarcity of reported mental health outcomes in relation to social well-being indicates that this area must be actively investigated to improve our understanding of its importance.

## 5 Discussion

In this review, we summarised current knowledge on the mental health of people receiving invasive home mechanical ventilation through a tracheostomy. We showed that within the time span from 2010 to 2022, only 13 studies (mainly published in medical journals) reported on mental health of people living with home mechanical ventilation in a home setting. Of these, seven studies have been published within the past six years, including five studies focusing directly on mental health. This review therefore adds to a field of research that seems to be new and sparsely studied, as the total number of included studies was rather small. That this research field has been sparsely investigated or is potentially difficult to locate is confirmed by Nakarada-Kordic et al. (2018), who examined, among other things, well-being and quality of life of adults living with tracheostomy in varying settings. Here, as in the present review, the researchers included only a small number of studies in a home setting (Nakarada-Kordic et al., 2018).

The majority of studies (*n* = 10; 76.9%) in the present review have been authored in Europe, followed by the Americas and the Western Pacific. This suggests that differences in healthcare systems and resources are mirrored in research output. This European and Western dominance in research within home mechanical ventilation and tracheostomy was also found in other reviews (MacIntyre et al., 2016; Nakarada-Kordic et al., 2018; Ørtenblad et al., 2019) and was therefore not a surprising finding of the present study. Attitudes towards treatment of tracheostomy-ventilated people vary worldwide (Garner et al., 2013; Kim et al., 2019; Lloyd-Owen et al., 2005; Rose et al., 2015; Simonds, 2016). In several parts of the world, home mechanical ventilation is not an option for the general population (MacIntyre et al., 2016; Masefield et al., 2017; van der Poel et al., 2017). Furthermore, in some of the studies, the number of participants receiving home tracheostomy ventilation was small, which may affect transferability and reliability. Therefore, we suggest conducting cross-country collaboration studies in this field to obtain a large number of participants. Such collaboration has previously produced relevant and useful knowledge in home mechanical ventilation (Farre et al., 2005; Lloyd-Owen et al., 2005).

In the present review, private home settings were widely investigated. In contrast, more specific settings like long-term care facilities or institutions were less widely investigated; presumably because the cost of maintaining mechanical ventilation at home is likely much lower than the cost of living in long-term care (MacIntyre et al., 2016). Another reason may be that users prefer to live at home (private) if they can. Based on our findings, we speculate that this may be so because living at home has a favourable effect on their emotional well-being. Therefore, we suggest studies measuring and comparing quality of life in various home settings to ensure that people get to live in their preferred settings. We found that quality of life across different home settings has been sparsely explored. This is surprising as quality of life is one of the most frequently-examined outcomes, also in relation to home mechanical ventilation, which has been confirmed by several international analyses (Brurberg and Dahm, 2014; Brurberg et al., 2012a; Brurberg et al., 2012b; MacIntyre et al., 2016; Ørtenblad et al., 2019). We therefore suggest that more comparative research on quality of life across care settings.

The present review gives insights into the users’ mental health based on a wide range of reported mental health outcomes in a setting in which no therapeutic alternatives exist and users would die from their illness without treatment. In relation to the sub-component *positive affect*, home mechanical ventilation gave people an interest in the lived life, which is confirmed by an international analysis by MacLaren et al. (2019). Moreover, MacLaren et al. (2019) reported that home mechanical ventilation does not by itself make it possible to live a satisfying life. We supported this conclusion by also finding that people receiving tracheostomy ventilation at home have a worrying life. Additionally, in their review, Nakarada-Kordic et al. (2018) found a significant reduction in life satisfaction among people living with tracheostomy ventilation. This finding contrasts with the findings in the present review where people in general and those who lived at home (private) in particular reported increased life energy and satisfaction with the changed life situation.

Moreover, we found that in relation to the sub-component *positive relations with others*, trusting relationships with family and personal assistants were important. These findings have also been reported in other studies (MacLaren et al., 2019; Schaepe and Ewers, 2017) and are further supported by a study by Markussen et al. (2018), who found that satisfaction with training in long-term mechanical ventilation and follow-up, delivered by professionals, improved ‘psychological well-being’ (p. 658). However, the positive implications of home mechanical ventilation can be far less for relatives, especially for those who are responsible for providing the care (Winther et al., 2020; MacLaren et al., 2019). Therefore, it would be interesting to examine the relatives’ and the personal assistants’ mental health status. This would be important also because both groups are important in relation to the user's psychological well-being, as seen in the sub-component *positive relations with others*, and social well-being, as seen in the sub-components of *social acceptance* and *social integration*.

Finally, we have shown that in relation to the sub-component *environmental mastery*, being able to handle the responsibilities in life that come with receiving home mechanical ventilation and care, was important to people. However, being profoundly disabled, such as those who are mechanically tracheostomy-ventilated at home, is associated with natural and expected limitations in terms of their opportunity to handle the responsibilities in life in a meaningful way on their own (Dreyer et al., 2010a; Briscoe and Woodgate, 2010; Dyrstad et al., 2013; Stuart and Weinrich, 2004). Therefore, these findings highlight the need for organised and individualised treatment and care for the people receiving this treatment (Stuart and Weinrich, 2004). The findings also highlight the need for mental health competencies among personal assistants to ensure successful collaboration (Dreyer et al., 2010a; Briscoe and Woodgate, 2010; Dyrstad et al., 2013) allowing tracheostomy-ventilated people to take on the desired responsibility. The need for personal assistants to develop and master mental health competencies is further substantiated by the findings in this review in relation to the sub-components *autonomy and social acceptance*. Thus, some people were seen to fight with health professionals and the healthcare system for their right to live autonomously, even if people's autonomy is a fundamental ethical principle to keep in mind in healthcare services (Greaney and O'Mathúna, 2017; Olejarczyk and Young, 2021).

### 5.1 Limitations

In this scoping review, the following limitations must be taken into consideration. Firstly, although a systematic search process was undertaken and an inclusive approach adopted, there is no guarantee that all eligible studies were identified. For example, potentially relevant studies could have been missed due to the search limitations relating to time span, although Arksey and O'Malley (2005) explicitly describe time span as a necessary decision to make from a practical point of view. Our desire was to ensure a contemporary knowledge base within a field undergoing significant development for which we believe that the time span used is relevant. Moreover, the search strategy was verified by a librarian to ensure quality despite the search limitations (Peters et al., 2020), which is a strength of this review. Secondly, one of the included studies was conducted by one of the researchers of this review (Dreyer et al., 2010b). This could have affected the assessment of its methodological quality (Logan et al., 2010; Pautasso, 2013); however, to counter this possibility (Pautasso, 2013), we had two other researchers (MLP and CH) make the assessment of this particular study, which is a strength of this review. Thus, we avoided the conflict of interest that arises when researchers have published relevant studies for inclusion in the review they write and the risk of bias that can occur in both directions where one's own research results are either overestimated or underestimated (Pautasso, 2013). Thirdly, this scoping review used the World Health Organization's (2004) definition of mental health. However, the definition of mental health is controversial; it has been discussed and has changed over time, and new definitions have been proposed (Galderisi et al., 2015; Keyes, 2014). Therefore, the use of a different definition of mental health may have led to selection of other studies, and thus other results may have emerged if another definition had been used.

### 5.2 Conclusions

In this review focusing on adults, we identified a heterogeneity of reported mental health outcomes, both positive and negative. Outcomes were mostly reported in relation to emotional well-being, which was followed by psychological well-being and social well-being. Emotional well-being was reported as an important mental health area by people receiving home mechanical ventilation through a tracheostomy, both in relation to the sub-components *positive affect* and *judgements of quality of life*. Psychological well-being and social well-being were reported with fragmented mental health outcomes according to the sub-components. Some of these sub-components were only sparsely investigated or not investigated at all. Therefore, mental health areas of psychological and social well-being must be actively researched if our understanding of these important dimensions is to be improved.

### 5.3 Perspectives

We showed that mental health in home tracheostomy ventilation is a sparsely investigated area; however, this is surprising as mental health problems are well-known and common among the users where cognitive abnormalities, behavioural disturbances, and psychiatric disorders are known in underlying diseases (Caspers Conway et al., 2015; Crockford et al., 2018; Peterson et al., 2020). As the underlying disease seems to predominantly influence health-related quality of life (Windisch and Criée, 2006), which we also found, this review further highlights the need to focus on the mental health of people receiving invasive home mechanical ventilation through a tracheostomy to improve our knowledge of this field. Here, research focusing on each component of well-being with clearly denied concepts and treatments is strongly recommended.

## Funding statement

No external funding.

## Declaration of Competing Interest

The authors declare that they have no known competing financial interests or personal relationships that could have appeared to influence the work reported in this paper.
